# Detection of Rice Fungal Spores Based on Micro- Hyperspectral and Microfluidic Techniques

**DOI:** 10.3390/bios13020278

**Published:** 2023-02-15

**Authors:** Xiaodong Zhang, Houjian Song, Yafei Wang, Lian Hu, Pei Wang, Hanping Mao

**Affiliations:** 1 School of Agricultural Engineering, Jiangsu University, Zhenjiang 212013, China; 2 Key Laboratory of Modern Agricultural Equipment and Technology, Ministry of Education, Jiangsu University, Zhenjiang 212013, China; 3 Key Laboratory of Key Technology on Agricultural Machine and Equipment, Ministry of Education, South China Agricultural University, Guangzhou 510642, China

**Keywords:** rice fungal spores, microfluidic chip, micro-hyperspectral, classification method

## Abstract

As rice is one of the world’s most important food crops, protecting it from fungal diseases is very important for agricultural production. At present, it is difficult to diagnose rice fungal diseases at an early stage using relevant technologies, and there are a lack of rapid detection methods. This study proposes a microfluidic chip-based method combined with microscopic hyperspectral detection of rice fungal disease spores. First, a microfluidic chip with a dual inlet and three-stage structure was designed to separate and enrich *Magnaporthe grisea* spores and *Ustilaginoidea virens* spores in air. Then, the microscopic hyperspectral instrument was used to collect the hyperspectral data of the fungal disease spores in the enrichment area, and the competitive adaptive reweighting algorithm (CARS) was used to screen the characteristic bands of the spectral data collected from the spores of the two fungal diseases. Finally, the support vector machine (SVM) and convolutional neural network (CNN) were used to build the full-band classification model and the CARS filtered characteristic wavelength classification model, respectively. The results showed that the actual enrichment efficiency of the microfluidic chip designed in this study on *Magnaporthe grisea* spores and *Ustilaginoidea virens* spores was 82.67% and 80.70%, respectively. In the established model, the CARS-CNN classification model is the best for the classification of *Magnaporthe grisea* spores and *Ustilaginoidea virens* spores, and its F1-core index can reach 0.960 and 0.949, respectively. This study can effectively isolate and enrich *Magnaporthe grisea* spores and *Ustilaginoidea virens* spores, providing new methods and ideas for early detection of rice fungal disease spores.

## 1. Introduction

Rice is a cereal belonging to the genus Oryza and is one of the most important food crops in Asia. It is the staple food for about half of the world’s population, 90% of which is produced in Asia [1,2]. Rice fungal disease can affect rice throughout the plant’s growth cycle, resulting not only in a large area of reduced or no rice yield, but also directly threatening the quality of rice seed [3]. Rice fungal diseases are mostly caused by fungal spores [4,5], which are small asexual propagules that are mainly airborne [6]. When airborne spores reach a certain number and find the right temperature and humidity, they germinate and reproduce rapidly. The more conidia, the wider the spread [7,8], especially for *Magnaporthe grisea* and *Ustilaginoidea virens*. Rice losses caused by *Magnaporthe grisea* are in the hundreds of millions of kilograms worldwide each year. *Magnaporthe grisea* is widespread and can occur throughout the rice growing season in almost all rice regions [9]. *Ustilaginoidea virens* usually occurs from the flowering to the milky stage of rice. Once *Ustilaginoidea virens* has invaded the grain, its incidence and rate of expansion are very rapid and ultimately lead directly to an increase in the rate of empty and shriveled rice. In addition, the rice grain mixed with *Ustilaginoidea* virens affects the quality of rice, which has become one of the three new diseases of rice [10]. As a result, knowing how to quickly capture and accurately identify spores at the early stage of rice fungal spore transmission is critical for early disease prediction.

Due to the complex composition of the air and the variety of microorganisms [11], spores and other microorganisms are suspended in the air stream in a constant state of collision and agglomeration. Therefore, the effective separation and capture of spores from the air become the primary issues for spore detection. Currently, spore collection instruments and air samplers are used to collect spores [12,13]. The collection efficiency of such instruments is low and there are additional losses in the process of capturing spores [14]. The concentration of captured spores is low, which is difficult to apply directly to the detection of airborne microorganisms. It is necessary to perform enrichment and purification before detection. In addition, a large amount of dust in the air can block the sampler, interfering with the collection and detection of target spores, and it is difficult to achieve continuous monitoring [15]. Air samplers often collect several target spores in the same collection box at the same time. It is difficult to capture two or more spores into different capture boxes in one capture, and it is difficult to achieve the effect of classification in the capture process [16]. Compared with traditional spore capture methods, microfluidic technology is a fast and high-throughput detection method that can realize automatic analysis, integration, miniaturization, and low consumption [17,18]. Lee et al. [19] designed a new microchannel with a double-arc unit structure, which can realize multiple filtrations of microparticles of different sizes, and the separation efficiency of the designed channel microfilter reaches 90.1%. Xu et al. [20] proposed a method to directly and accurately extract microorganisms from the air flow based on a microfluidic chip. The chip achieved high-purity extraction of mold spores and gray mold spores, with extraction rates of 89% and 76%, respectively. Microfluidics relies on the key advantages of microchannel miniaturization to develop a variety of new applications that are shining in areas such as biomedicine, food testing, and environmental monitoring [21,22].

The ultimate purpose of capturing fungal spores is to detect them, and common spore detection methods include polymerase chain reaction (PCR), image recognition, etc. [23,24]. Araujo et al. [25] used a highly specific qPCR technique to identify and quantify the airborne inoculum of six Canadian wheat pathogens in real time, and developed a rapid and reliable prediction system to identify and quantify airborne pathogens in real time before the onset of disease symptoms. Aguayo et al. [26] used high-throughput sequencing to perform real-time fluorescent PCR against eight forest pathogens, of which five out of eight were detected by real-time PCR, and the spatial and temporal trends of pathogen detection were consistent with field data. Although the PCR detection method is a standard method for detecting microorganisms with high sensitivity and accuracy [27], it requires specific antibodies or primers and a harsh detection environment, otherwise it is easy to produce false positive results due to environmental contamination, and it is difficult to perform real-time detection in complex environments [28]. Due to the small size and similar shape of rice fungal spores, when the image recognition detection method is used to detect fungal spores, it is easy to confuse them with other contaminants in the environment, and it is difficult to extract morphological information [29]. Although the above spore detection technology has been proven to be feasible, the specific antibody is difficult to obtain and the operation is relatively complex, and has not been widely used in practice. At present, the conventional laboratory detection method is still mainly used, that is, the inspector will observe the collected samples through the optical microscope and complete the detection according to the morphological characteristics of the spores under the microscope [30]. The use of manual detection methods is time-consuming and laborious, and it can easily cause large errors. Therefore, in order to improve the accuracy of airborne spore detection, it is necessary to study simpler and efficient spore detection technology.

Microscopic hyperspectral imaging technology combines the advantages of hyperspectral and microscopic technologies. It can perform non-invasive detection of microorganisms at the cell level, and has the advantages of rapid and non-destructive detection. It can not only collect the images of small targets, but also collect the spectral information of the target area at the same time. According to the theory of light transport in biological tissues, the optical properties of living tissues, such as absorption, radiation, reflection, and anisotropy, depend on their biochemical composition and morphological structure. The specific spectra contained in different cells and tissues are like fingerprints, containing subtle differences, and can therefore be used to represent and distinguish objects [31,32], and greatly reduce the workload of testers. In recent years, hyperspectral imaging technology (HSI), originally used in remote sensing, has been extended to the biological field [33]. Sun et al. [34] developed a fungal growth simulation model based on HSI, and the classification accuracy of the model test data set reached 97.5%, providing a new idea for identifying fruit decay caused by fungi. Zhang et al. [35] used SWIR-HSI to predict the morphological structure and compositional changes in aflatoxin-infected peanut kernels, demonstrated significant differences in the infrared absorption peaks of nutrients and fungal toxins in healthy and unhealthy peanut kernels, and proposed a new method for detecting aflatoxin in peanut kernels. It can be seen that hyperspectral imaging technology has been put into practice in fungus detection research.

Therefore, this study proposed a method based on a microfluidic chip combined with the microscopic hyperspectral detection of rice fungal disease spores. A microfluidic chip is designed for *Magnaporthe grisea* spores and *Ustilaginoidea virens* spores, enabling the chip to separate other particles in the air and collect the two types of spores in corresponding enrichment areas, combined with microscopic hyperspectral imaging technology to detect the spores and establish a classification model based on the spectral characteristics of the spores, providing a new method and new ideas for the early detection of spores of rice fungal diseases.

## 2. Materials and Methods

### 2.1. Spore Sample Preparation

The strains of *Magnaporthe grisea* fungus and *Ustilaginoidea virens* balls were used to prepare the spore suspension were provided by the China National Rice Research Institute. The pathogen of *Magnaporthe grisea* fungus was inoculated on the slant culture of potato dextrose agar (PDA) slant medium and cultivated in a sterile environment with temperature of 28 °C and humidity of 85% RH. After 10 days of incubation, a small amount of sterile distilled water was added to the PDA slant culture medium, and the fresh *Magnaporthe grisea* spores on the surface were gently scraped with a sterile inoculation ring to obtain the spore suspension. However, at this time, there were some impurities in the suspension, which we filtered with a single layer of sterile medical cotton gauze to remove the mycelium. The filtered suspension was placed in a sterile centrifuge tube, the spores were separated and precipitated by centrifugation, and finally the concentration of the spore suspension was determined using a blood cell counting plate and adjusted to 5 × 10^6^ spores/mL, with 10 mL of the prepared suspension of *Magnaporthe grisea* spores being taken as the experimental sample. The ball of *Ustilaginoidea virens* is the diseased grain that develops after *Ustilaginoidea virens* has been infected by Aspergillus oryzae. There is a large amount of chlamydospore layer on its surface, so it is not necessary to culture it in PDA slant medium. The chlamydospore on the surface of the *Ustilaginoidea virens* ball can be scraped directly into sterile water to obtain the spore suspension, then filtered through a single layer of sterile gauze several times, and centrifuged to obtain a concentration of 5 × 10^6^ spores/mL suspension of *Ustilaginoidea virens* spores.

### 2.2. Working Theory of Microfluidic Chip

Spore movement in microchannels is a sparse two-phase flow with a low Reynolds number [36]. The Reynolds number $\left(Re\right)$ is the ratio of the inertial force to the viscous force acting on each micro-element of the fluid in a microfluidic chip, and is a dimensionless number describing the fluid flow condition. The Reynolds number is expressed as [37]

(1) $$Re=\frac{\rho vd}{\eta }$$

where ρ,v,η are, respectively, the density (kg/m^3^), velocity (m/s), and viscosity coefficient of air flow (Pa·s); d is the characteristic length of air flow (m).

When the airflow bypasses an obstacle and suddenly changes direction, the airflow exhibits a curvature. Suspended spores move in a curved pattern along the streamline as the airflow changes [38]. The motion profile of spores within the microchannel of a microfluidic chip can be described by the Stokes number $(S_{tk}$), which is given by [39]

(2) $$S_{tk}=\frac{t_{0}u_{0}}{L_{0}}$$

where $t_{0}$ is the relaxation time of the spore (s), $u_{0}$ is the flow velocity of the gas as it passes through the obstacle (m/s), and $L_{0}$ is the characteristic size of the obstacle (m). When $s_{tk}$ > 1, the streamline bypasses the obstacle while the particle still travels in its initial direction, resulting in an impact on the obstacle; when $s_{tk}$ ≤ 1, the particle follows the streamline and undergoes a deflection.

Rice fungal disease spores accelerating in the gas will drive the surrounding gas to accelerate; this effect is equivalent to the spores having additional mass. The additional mass force $\left(F_{t}\right)$ expression is [40]

(3) $$F_{t}=\frac{1}{12}d_{p}^{3}\rho \frac{d}{dt}\left(v-v_{p}\right)$$

where $d_{p}$ is the spore particle size (m), ρ is the gas density (kg/m^3^), v is the gas velocity vector (m/s), and $v_{p}$ is the spore velocity vector (m/s).

### 2.3. Structure Design and Fabrication of Microfluidic Chip

#### 2.3.1. Structure Design of Microfluidic Chip

**Figure 1:**
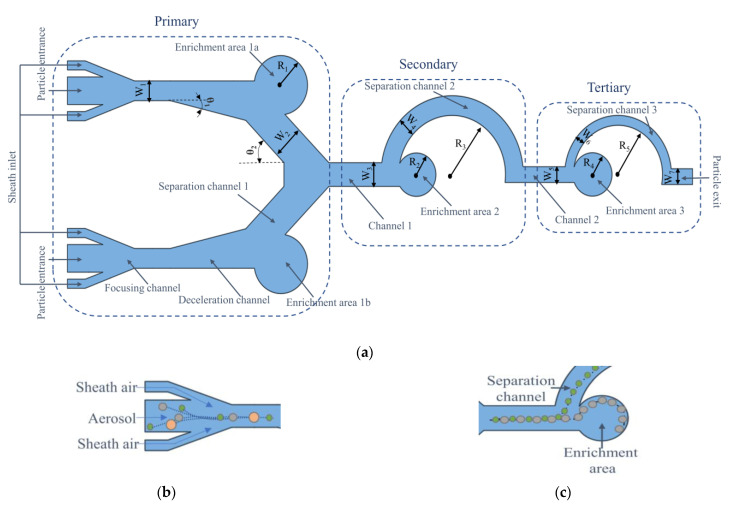
(**a**) The two-dimensional structure of the microfluidic chip. The enrichment areas 1a and 1b in the chip collect particles with larger particle size in the air, enrichment area 2 collects *Magnaporthe grisea* spores, enrichment area 3 collects *Ustilaginoidea virens* spores, and particles with smaller particle size are discharged from the outlet. (**b**) Schematic diagram of sheath flow working principle; (**c**) schematic diagram of separation channel working principle.

The two-dimensional structure of the microfluidic chip designed in this study is shown in Figure 1a. The chip can be divided into three structures, each with a corresponding separation channel and enrichment area. The first stage structure is designed as a double inlet symmetrical pre-treatment channel, where the airborne spores first enter the first stage structure through the inlet and pass through the sheath flow channel. The main function of the sheath flow channel is to squeeze the airflow containing the spores into the microchannel, ensuring that the spores entering the chip form a single spore flow array in the center of the microchannel, thus allowing the spores to be focused [41]. As the sheath flow channel also causes an increase in the velocity and density of the gas in the flow channel, the additional mass force on the spores increases and some of the spores will hit the chip walls directly without moving with the airflow. Therefore, a deceleration channel is added after the sheath flow channel to reduce the effect of additional inertial forces on the movement of the spores. Spores in the channel are also affected by inertia when moving with the airflow [42]. Spores of smaller mass and size will move with the airflow, enter the separation channel, and reach the next level of the structure for separation; while, spores of larger mass and size will continue to move in the original direction due to inertia, and eventually will rush into the enrichment area. The operating principles of the chip sheath flow and the separation channel are shown in Figure 1b,c, respectively.

#### 2.3.2. Fabrication of Microfluidic Chip

**Figure 2:**
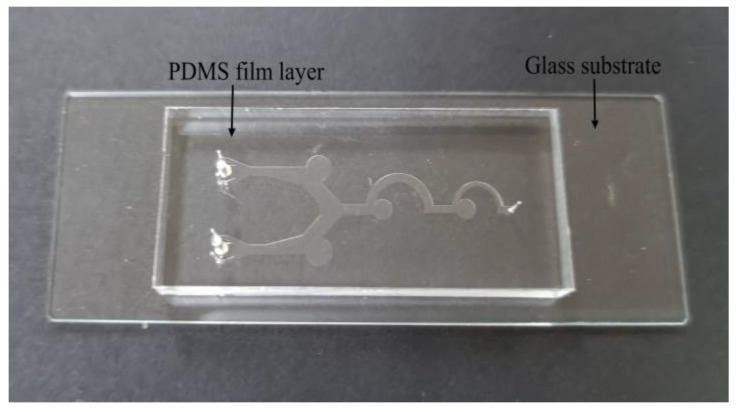
Real object of microfluidic chip.

The choice of materials and processing technology for the microfluidic chips plays a very important role in the performance of the chip itself. The PDMS polymer has become the most commonly used material for microfluidic chips due to its high optical transparency, strong plasticity, and easy combination with other substrates [43]. In this study, the standard soft lithography method is used to fabricate the designed chip. The fabrication equipment is simple, and more complex microfluidic chips can be fabricated [44]. The fabrication requires polishing the copper plate, microchannel film, photosensitive dry film, developer, PDMS solution, casting mold, tape, knife, and other materials. A 100 μm thick photosensitive dry film is placed on the polished copper plate, and then the microchannel film is placed as a mask tightly over the photosensitive dry film with one side of the photosensitive dry film facing down, and is placed in the exposure machine for exposure. After the exposure is completed, the copper plate, from which the microchannel film is torn, is placed in the prepared developer for development, and then the casting mold is placed on the copper plate according to the position of the microchannel after development. The mixed PDMS solution is poured into the center of the casting mold, and the PDMS solution is placed in the vacuum pump. After the bubbles in the PDMS solution overflow, it is placed in the electric blast drying oven at a temperature of 6 °C to dry for 5 h, and then it is taken out. Then, the PDMS is carefully removed from the mold. The glass substrate is bonded with the side of the PDMS using a channel, and it is pressed gently with a low-dust wiping paper. Finally, the bonded chip is subjected to a 60 °C constant temperature heating stage, waiting for 15 min for the fabrication to be complete. Figure 2 shows the actual image of the microfluidic chip prepared in this study.

### 2.4. Numerical Simulation Analysis

#### 2.4.1. Parameter Setting

This study uses Soildworks 2021 (Dassault Systemes, Concord, MA, USA) mapping software to draw the two-dimensional structure of a microfluidic chip. In the channel design, the sheath flow channel width W_1_ is preset to 1000 μm; θ_1_ is set to 15°. Enrichment area 1a radius R_1_ is 1500 μm, the same radius as enrichment area 1b. Enrichment area 2 radius R_2_ is the same size as the enrichment area 3 radius R_4_; both are 1100 μm. The semi-circular separation channel R_3_ is set to 3000 μm; R_5_ is set to 2500 μm; exit channel width W_7_ is 800 μm; all microchannels are 100 μm thick. Numerical simulation of the flow field and spore motion of a microfluidic chip use COMSOL Multiphysics 6.0 multiphysics simulation software (COMSOL Inc, Stockholm, Sweden) to validate and optimize the chip parameters. In COMSOL Multiphysics 6.0, the particle tracking module sets the simulation parameters to a uniform distribution of 100 identical particles at the entrance of each microfluidic chip. Since the microfluidic chip designed for this study is a dual inlet, a total of 200 particles enter the chip simultaneously for each simulation. To simulate the flow rate at the chip inlet and the sheath flow, the particle inlet flow rate is set to 12.5 mL/min, the flow rate at each sheath flow inlet is set to 2.5 mL/min, and the particle density is set to 1000 kg/m^3^. As the wall of microfluidic chip is an inelastic body, the spores will not slide when they collide with the wall, and most of the spores will adhere to the wall. Therefore, when the spores collide with the wall of the microchannel, the viscosity is 95% and the remaining 5% is rebound.

#### 2.4.2. Simulation Optimization

Many of the parameters in the chip designed for this study are still undetermined in size because they have been found to have a large impact on the particle enrichment effect after simulation, and therefore need to be numerically optimized for selection. The magnitude of the θ_2_ and W_2_ values affects the enrichment effect in enrichment areas 1a and 1b, and the values of W_3_/W_4_ and W_5_/W_6_ affect the enrichment effect in enrichment area 2 and enrichment area 3, respectively. By varying the range of values for θ_2_, W_2_, W_3_/W_4_, and W_5_/W_6_, the enrichment efficiency of the corresponding enrichment area is changed and the value of the parameter with the highest enrichment efficiency is selected. The enrichment efficiency of the particles can be calculated according to Equation (4) [11].

(4) $$\eta =\frac{\eta_{i}}{\eta_{j}}\times 100\%$$

where $\eta_{i}$ is the number of particles of a given particle size entering the enrichment area; $\eta_{j}$ is the total number of particles of a given particle size released into the chip.

Through observing different batches of spores cultured at different stages with an ultra-deep three-dimensional microscope, it was found that the grain size of *Ustilaginoidea virens* spores is generally 4 μm to 6 μm, and the diameter of *Magnaporthe grisea* spores is generally 7 μm to 17 μm. In order to have better simulation results, the simulations were carried out with 5 μm particles to characterize *Ustilaginoidea virens* spores, 12 μm particles to characterize *Magnaporthe grisea* spores, 25 μm particles to characterize larger impurities in the air, and 2μm particles to characterize smaller impurities in the air.

### 2.5. Micro-Hyperspectral Data Collection and Processing Analysis of Fungal Disease Spores

#### 2.5.1. Acquisition of Micro-Hyperspectral Data of Fungal Disease Spores

The GaiaMicro series microscopic hyperspectral imaging system built by Jiangsu Dualix Spectral lmaging Technology Company (Wuxi, China) was used in this study, which mainly consists of a microscope, an AOTF device, a charge-coupled device detector (CCD), a data acquisition module, and a computer. The effective wavelength range of the equipment spectrum is 400–1000 nm, the spectral resolution is 2.8 nm, and the spectral sampling rate is 0.7 nm. After the PDMS film layer of the chip is removed after enrichment, it is placed under the microscopic hyperspectral to find the position of fungal disease spores through the 40× objective lens, and the focus of the microscope is finely adjusted to ensure that the spores are in focus at the microscope eyepiece and CCD camera. Spore images at different positions are collected by controlling the moving field of view of the precise electric control translation mechanism. Combined with the AOTF device, we obtained the cubic hyperspectral data of spores in 120 wavebands in the wavelength range of 400–1000 nm. As microscopic hyperspectral imaging systems are susceptible to dark currents and light intensity, black and white correction is required to minimize the effect of ambient noise [45]. Before spore data collection, the spectral curve of the spectrometer on the polyvinyl chloride (PVC) white board and in the dark environment was collected to correct the micro-hyperspectral image data. The correction formula is shown in Formula (5) [45].

(5) $$R=\frac{R_{i}-R_{b}}{R_{w}-R_{b}}$$

where R is the corrected hyperspectral image data; $R_{i}$ is the original hyperspectral image data; $R_{w}$ is the PVC whiteboard calibration image data; and $R_{b}$ is the dark environment calibration image data.

#### 2.5.2. Extraction of Micro-Hyperspectral Data of Fungal Disease Spores

In this study, the hyperspectral images of the spores were opened using ENVI 5.3 hyperspectral image processing software, and an area of 15 × 15 pixels in the center of each spore was selected as the region of interest by selecting of the rectangle tool in the region of interest (ROI) toolbar of the software. For each spore species, 15 hyperspectral images were collected; 10 clearly photographed spores in each image were selected for ROI extraction, and the average of the spectral reflectance of all pixels in each ROI was calculated as the spectral data of this sample. In total, 300 spectral data were obtained for the two spores.

#### 2.5.3. Selection of Characteristic Wavelength of Micro-Hyperspectral of Fungal Disease Spores

There is a large amount of spectral information in the full band, and redundant information interferes with the accuracy of the classification mode. Inputting each spectral band into the model for analysis will increase the amount of model operations, reduce the speed of model operations, and make it difficult to meet the requirements of rapid detection. Therefore, it is necessary to filter out the characteristic waves of the spectrum to effectively simplify the model. Competitive adaptive reweighted sampling (CARS) is a common algorithm used to filter optimal feature combinations [46]. It is a wavelength selection method based on partial least squares regression coefficients, which selects the variable points with the largest absolute value of the regression coefficient, removes the points with smaller weight values, and filters the subset of bands with the smallest RMSECV values. The variables contained in the subset of bands are the optimal variable combinations.

#### 2.5.4. Classification Mode

In this study, Support Vector Machine (SVM) and Convolution Neural Network (CNN) were compared to select the best model for the classification of rice blast spores, and rice aspergillus SVM is a typical supervised classifier and a two-class classification model. Its basic model is defined as a linear classifier with the largest interval in the feature space. The learning strategy is to maximize the interval, which can ultimately be transformed into a convex quadratic programming problem [47]. SVM can find the best method between the complexity of the model and the learning ability according to the limited sample information, in order to obtain the best generalization ability. CNN is a feedforward neural network that includes both convolutional computation and depth structure. The essence of its learning process is to extract the characteristics of input data by establishing multiple filters [48]. The CNN model in this study includes an input layer, a full connection layer, a normalization layer, and an output layer. The convolutional layer, batch norm layer, activation layer, and pooling layer are two each, where the convolutional kernel size is 11 × 11 and 9 × 9 with the numbers of 16 and 32, respectively, and the pooling layer size is 11 × 11 and 8 × 8, respectively, using the maximum pooling function.

### 2.6. Classification Evaluation Indicators

In order to more intuitively show the classification effects of different models, this study uses the classification performance indicators of accuracy, precision, recall, specificity and F1-Core to express effects. The calculation formula is Formulas (6)–(10) [49].

(6) $$Accuracy=\frac{TP+TN}{TP+TN+FP+FN}$$

(7) $$Precision=\frac{TP}{TP+FP}$$

(8) $$Recall=\frac{TP}{TP+FN}$$

(9) $$Specificity=\frac{TN}{TN+FN}$$

(10) $$F1-Score=\frac{2*Precision*Recall}{Precision+Recall}$$

where *TP* (True Positive) is a positive sample predicted by the model to be positive; *FP* (False Positive) denotes a negative sample predicted to be positive by the model; *FN* (False Negative) indicates a positive sample predicted by the model to be negative; *TN* (True Negative) indicates a negative sample predicted by the model.

## 3. Results and Discussion

### 3.1. Microfluidic Chip Numerical Simulation Results

**Figure 3:**
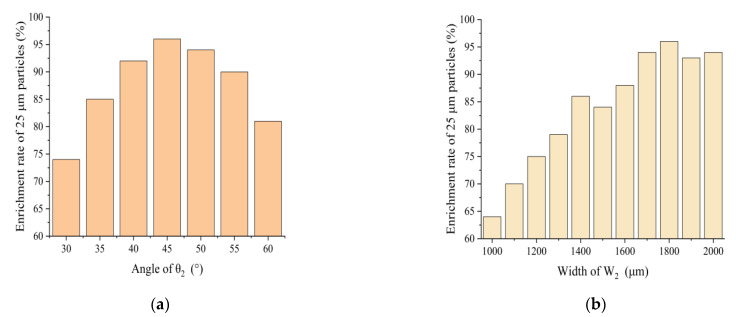
(**a**) Effect of θ_2_ on enrichment efficiency of 25 μm particles; (**b**) effect of W_2_ on enrichment effect of 25 μm particles.

In order to find the best value for the hidden parameter and to ensure that each enrichment area has the highest enrichment efficiency, different ranges of values were used for the simulation of the hidden parameter. Figure 3a,b show the channel angle θ_2_ and width W_2_ and 25 μm, and the relationship between enrichment efficiency of m particles in enrichment areas 1a and 1b. By choosing different values of θ_2_, it was found that the enrichment efficiency of 25 μm particles was relatively high when θ_2_ was set to 45°. The relationship between the width of W_2_ for 25 μm particles is shown in Figure 3b. The highest enrichment efficiency of 96% was achieved when W_2_ was set to 1800 μm, with θ_2_ finally chosen to be 45° and W_1_ to be 1800 μm.

**Figure 4:**
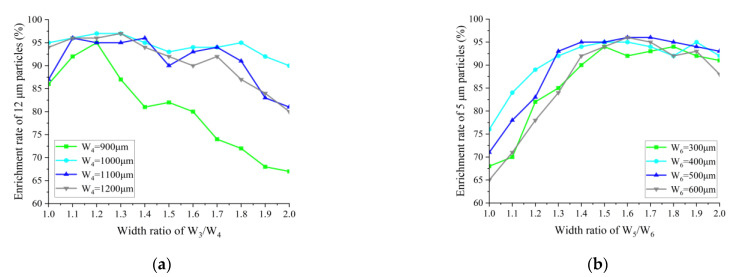
(**a**) Effect of W_3_/W_4_ on the enrichment efficiency of 12 μm particles; (**b**) effect of W_5_/W_6_ on the enrichment efficiency of 5 μm particles.

Figure 4a,b show the relationship between the enrichment efficiency of 12 μm particles in enrichment area 2, and 5 μm particles in enrichment area 3 for different W_3_/W_4_ and W_5_/W_6_ ratios, respectively. The highest enrichment efficiency of 97% for 12 μm is achieved when the width of W_4_ is 1000 μm and the width ratio of W_3_/W_4_ is 1.2 and 1.3; while, 97% is also achieved when the width of W_4_ is 1200 μm and the width ratio of W_3_/W_4_ is 1.3. In order to reduce the size of the chip as much as possible, a smaller channel width was chosen, so W_4_ was finally chosen to be 1000 μm, and W_3_/W_4_ was 1.2, at which point W_4_ was 1000 μm. At a W_5_/W_6_ width ratio of 1.6 and a W_6_ width of 500 μm or 600 μm, the maximum enrichment efficiency for particles of 5 μm is 96%. As there are inevitable deviations in channel widths in the actual fabrication of microfluidic chips, it is necessary to choose a relatively stable width for enrichment efficiency. Comparing the enrichment rates for W_6_ of 500 μm and 600 μm at ratios around a W_5_/W_6_ ratio of 1.6, it was found that the enrichment efficiency of ratios around a W_5_/W_6_ ratio of 1.6 decreased significantly faster at a W_6_ of 600 μm than at a W_6_ of 500 μm. Therefore, a W_6_ of 500 μm and a W_5_/W_6_ of 1.6 were chosen, at which point W_5_ was 800 μm.

**Figure 5:**
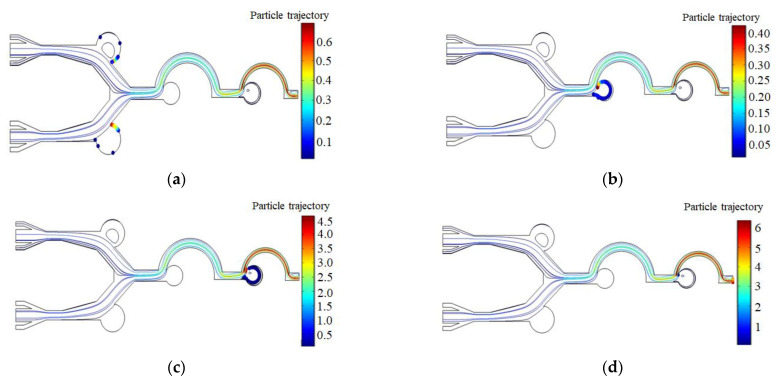

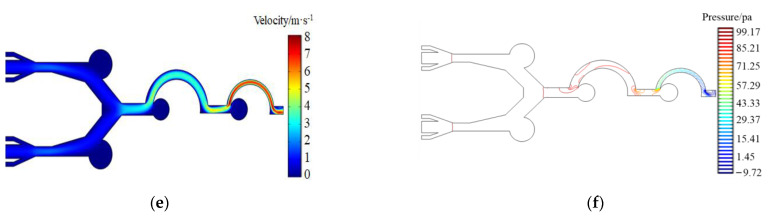
(**a**) Enrichment areas 1a and 1b for 25 μm particles; (**b**) enrichment area 2 for 12 μm particles; (**c**) enrichment area 3 for 5 μm particles; (**d**) exclusion of 2 μm particles from the outlet; (**e**) microfluidic chip channel velocity distribution; (**f**) microfluidic chip pressure distribution.

Figure 5a–d show the simulation of the enrichment effect of microfluidic chips designed in this study on particles of different sizes. The particles enter the chip from the inlet channel, and enter the sheath flow channel after receiving the initial rightward horizontal force. The sheath flow arranges the particles one at a time along the center line of the channel. Particles with a particle size greater than or equal to 18 μm will rush into the enrichment area 1a and 1b due to inertia, while particles with a particle size less than 18 μm will migrate with the airflow into the secondary structure, and particles with a particle size between 8 μm and 17 μm will rush into enrichment area 2. The third stage of the separation and enrichment structure is similar to the second stage, with particles with a particle size between 4 μm and 7 μm entering enrichment area 3, while particles with a particle size less than 4 μm being discharged directly from the outlet. The velocity distribution diagram of the microfluidic chip channel is shown in Figure 5e, and the pressure distribution intensity diagram of the microfluidic chip is shown in Figure 5f.

### 3.2. Results of Microfluidic Chip Spore Enrichment Experiments

**Figure 6:**
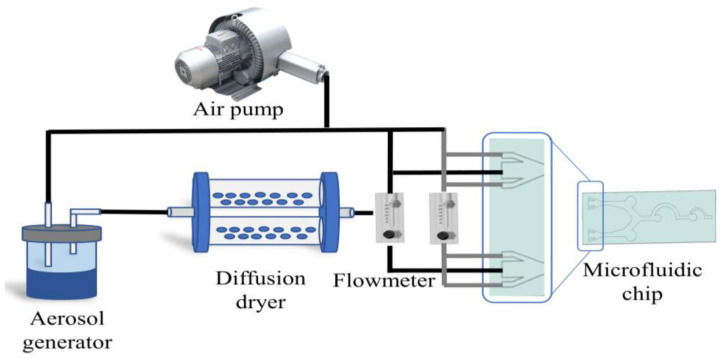
The experimental platform for spore enrichment.

Figure 6 shows the experimental platform for spore enrichment. The prepared spore suspension is placed in the aerosol generator, and the air is compressed by an air pump and fed into the aerosol generator to produce a bioaerosol stream. The aerosol stream passes into the diffusion dryer to remove the water. A flow meter (measuring range 2.5–25 mL/min) is connected after the dryer and the flow rate is set to 12.5 mL/min, the same value as in the chip simulation. At the same time, another microflow meter was connected to each sheath inflow port, and the sheath inflow port flow rate was set to 2.5 mL/min. The final aerosol flow entered the channels of the microfluidic chip to separate and enrich the spores. The spore enrichment experiment is completed after the air pump has been turned on for 2 min. After enrichment, the PDMS film is clamped with tweezers and removed slowly. If the PDMS is too tightly bonded to the substrate, a knife is used to assist. The microfluidic chip with the PDMS layer removed is placed under the microscope hyperspectrometer for direct observation and detection.

**Figure 7:**
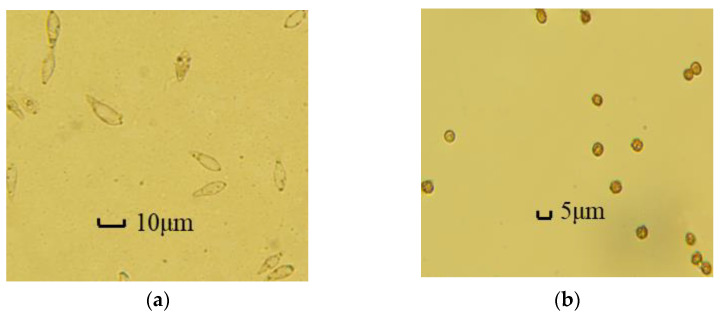
(**a**) Results of *Magnaporthe grisea* spores enrichment; (**b**) results of *Ustilaginoidea virens* spores enrichment.

Figure 7 shows the images collected by micro-hyperspectral in enrichment area 2 and enrichment area 3, respectively. Figure 7a shows the distribution of *Magnaporthe grisea* spores in enrichment area 2. It is found that a small amount of extremely small particles still attach to *Magnaporthe grisea* spores and enter the enrichment area together, but they have a good purification effect; Figure 7b shows the distribution of *Magnaporthe grisea* spores in enrichment area 3, and the *Magnaporthe grisea* spores can be more evenly distributed in the enrichment area.

To verify the actual enrichment efficiency of microfluidic chips, five microfluidic chips were obtained after enrichment through five spore enrichment experiments. First, the center and edge of four enrichment areas in each microfluidic chip were observed through a microscope, and the number of two kinds of spores in each enrichment area was counted. Then, the average number of two kinds of spores in each enrichment area of the five experiments was obtained, and the number of two kinds of spores in other channels was counted. Finally, the actual enrichment efficiency of the microfluidic chips for the two types of spores was obtained. It can be seen from Table 1 that the actual enrichment efficiency of the microfluidic chip designed in this study for *Magnaporthe grisea* spores is 82.67%, and the actual enrichment efficiency for *Ustilaginoidea virens* spores is 80.70%.

### 3.3. Spectral Data Analysis Results

**Figure 8:**
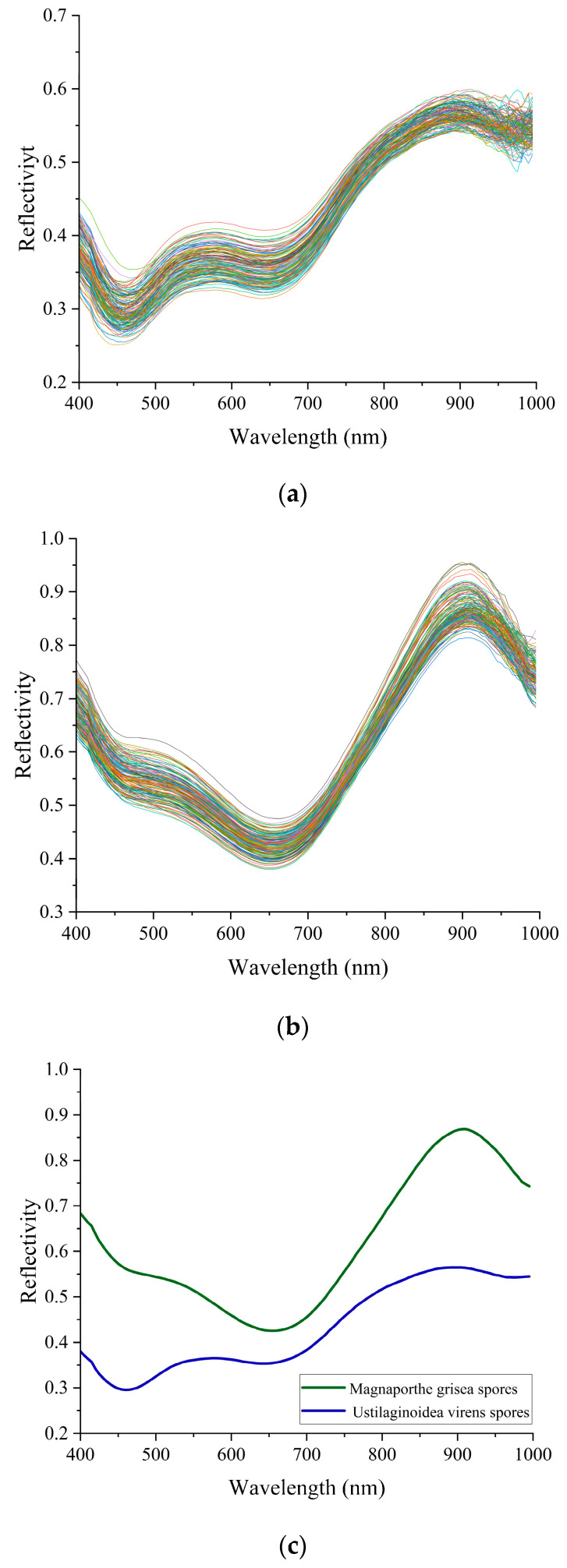
(**a**) is the original spectrum of *Magnaporthe grisea* spores. It can be roughly known that there are obvious troughs at the wavelength of 400 nm and 650 nm, and there are peaks at the wavelength of 550 nm and 900 nm; (**b**) is the original spectrum of *Ustilaginoidea virens* spores. We can see that there are wave troughs and peaks at the wavelengths of 650 nm and 900 nm, and the reflectivity rises sharply from the wave troughs, and basically rises to the highest point near 900 nm, forming a high reflection point. The rising speed and reflectivity intensity in this range are higher than that of rice aspergillosis spores; (**c**) is the average spectrum of spores.

According to the original spectra and treated spectra of *Magnaporthe grisea* spores and *Ustilaginoidea virens* spores, Figure 8a,b are the original spectra of *Magnaporthe grisea* spores and *Ustilaginoidea virens* spores, respectively, and Figure 8c is the average spectrum of the two types of spores. The spectral characteristics of the two spores can be seen to be distinct, with different characteristics in the 400–1000 nm wavelength range. Compared to *Ustilaginoidea virens* spores, the reflectance of *Magnaporthe grisea* spores is generally higher, which may be due to the strong absorption of light by pigments in *Ustilaginoidea virens* spores. There is a distinct wave peak near 550 nm for the *Ustilaginoidea virens* spores. The reflectivity continues to increase between 650–900 nm, and begins to decrease after 900 nm. Although the reflection curve of *Magnaporthe grisea* spores is similar to that of *Ustilaginoidea virens* spores in the range of 650–1000 nm, the rise and fall rate of rice blast spores is much faster than that of *Magnaporthe grisea* spores. Although the average spectrum of spores can reflect certain rules, it cannot represent the information of all samples due to the large individual differences in biological samples. Therefore, these spectral differences can only be used for preliminary qualitative analysis and cannot be used directly for identification. Further analysis of spectral data is required.

### 3.4. Characteristic Band Screening Results

**Figure 9:**
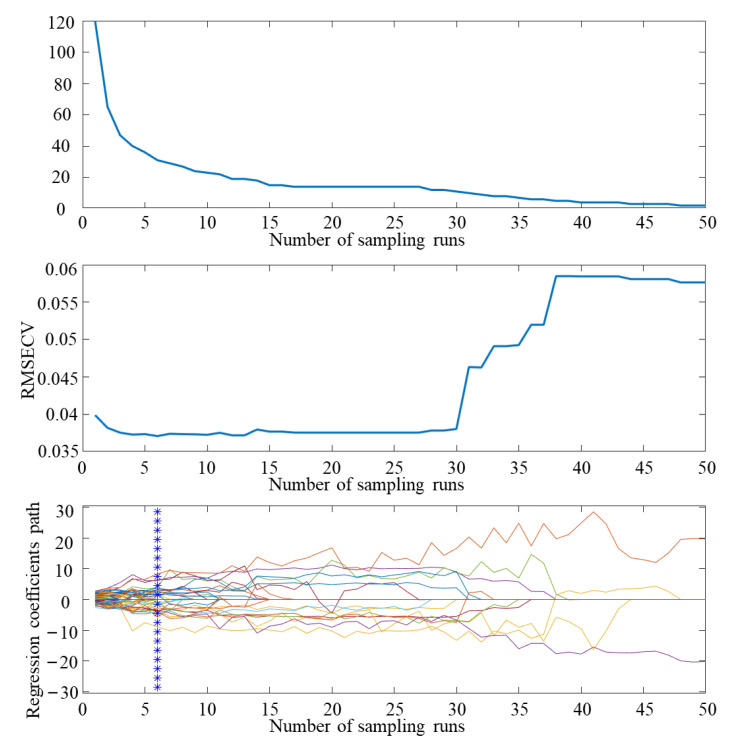
CARS feature screening results.

In this study, when using the CARS algorithm for screening, the number of round-robin samples was set to 50 and the results of CARS feature screening are shown in Figure 9. Analysis of the data shows that the overall decrease in the number of bands was retained as the number of iterations of the CARS algorithm increased, but at a sharp to slow rate, which was due to the change in the CARS algorithm from coarse to fine screening during feature band screening. From the change trend of the RMSECV value, it can be seen that as the number of samples increases, the RMSECV value tends to decrease. When the number of samples is six, the REMSECV value is the minimum, and the selected feature band subset is the best. A total of 34 feature wavelengths are selected (400.00, 404.89, 430.29, 450.10, 455.10, 475.00, 515.00, 530.00, 535.09, 545.09, 574.79, 585.00, 590.00, 625.00, 630.09, 669.79, 675.00, 680.09, 689.70, 709.70, 720.09, 744.70, 760.29, 779.79, 785.09, 800.09, 830.29, 855.29, 859.90, 869.79, 890.2, 894.90, 914.90 and 924.79 nm), accounting for 5.67% of the total band.

### 3.5. Classification Model Comparison Results

In this study, SVM and CNN classification algorithms are used to model the full band and feature band, respectively. One hundred groups of data were randomly selected from each spore in the dataset, and the training and test set were divided 8:2. Table 2 shows the classification results of the SVM classification model, the CNN classification model, and the combined model of these two models with the CARS algorithm for both spores. The table shows the values of TP, FN, TN, and FP obtained by the four classification models for the two spore species. Table 3 shows the four secondary indicators accuracy, precision, recall, and specificity, and the tertiary indicator F1-Score that were calculated. The classification model based on CARS-screened spectrum as a variable is better than the classification model based on full spectrum. By comparing the CARS-SVM classification model and CARS-CNN classification model, it is found that the two models have similar classification results for *Magnaporthe grisea* spores. However, among the classification indexes of *Ustilaginoidea virens* spores, the secondary indexes of the CARS-CNN classification model, accuracy, precision, recall, and specificity are 0.960, 0.950, 0.970, and 0.950, respectively, and the tertiary index F1-Score is 0.960, much higher than the CARS-SVM classification model. As the F1-Score index is closer to the numerical value 1, it means the classification effect is better; so, the CARS-CNN classification model has more advantages in classifying rice false smut spores. A comprehensive comparison of the CARS-CNN model has a better classification effect.

## 4. Conclusions

The purpose of this study is to propose a method for early and rapid detection of rice fungal disease spores, and to design a microfluidic chip with a dual inlet and three-stage structure to classify and enrich *Magnaporthe grisea* spores and *Ustilaginoidea virens* spores in the air. The enrichment results show that the enrichment rates of the microfluidic chip on *Magnaporthe grisea* spores and *Ustilaginoidea virens* spores are 82.67% and 80.70%, respectively. Combined with the hyperspectral image data of two kinds of spores collected using microscopic hyperspectrum, four classification models were established and compared. The results showed that the F1-Score indexes of *Magnaporthe grisea* spores and *Ustilaginoidea virens* spores in the CARS-CNN classification model were 0.949 and 0.960, respectively, which were more effective than the other three models. In addition, this study can be applied to the detection and classification of other fungal disease spores, providing a new method for the enrichment and detection of fungal spores. In the future, microfluidic chips and microscopic hyperspectrum will continue to be used to estimate the early spore concentration and disease severity of rice fungal diseases, and to quantify the spore concentration. With the application of fast, online, accurate, and cheap special sensors, portability and automation are bound to become the development trends of spore detection instruments. However, this road is still full of opportunities and challenges, and we must continue our efforts.

## Figures and Tables

**Table 1:** Statistical results of spore enrichment experiments.

Spore Type	Average Number of Spores Enriched				Sum	Enrichment Efficiency
	Enrichment Area 1a and 1b	Enrichment Area 2	Enrichment Area 3	Other Channels		
*Magnaporthe grisea* spores	15	291	4	42	352	82.67%
*Ustilaginoidea virens* spores	3	6	301	63	373	80.70%

**Table 2:** Classification results of different models.

Index	SVM		CNN		CARS-SVM		CARS-CNN	
	*Magnaporthe grisea* Spores	*Ustilaginoidea virens* Spores	*Magnaporthe grisea* Spores	*Ustilaginoidea virens* Spores	*Magnaporthe grisea* Spores	*Ustilaginoidea virens* Spores	*Magnaporthe grisea* Spores	*Ustilaginoidea virens* Spores
TP	84	82	83	87	93	94	94	97
FN	16	18	17	13	7	6	6	3
TN	82	83	85	84	95	91	96	95
FP	18	17	15	16	5	9	4	5

**Table 3:** Results of different model classification indicators.

Index	SVM		CNN		CARS-SVM		CARS-CNN	
	*Magnaporthe grisea* Spores	*Ustilaginoidea virens* Spores	*Magnaporthe grisea* Spores	*Ustilaginoidea virens* Spores	*Magnaporthe grisea* Spores	*Ustilaginoidea virens* Spores	*Magnaporthe grisea* Spores	*Ustilaginoidea virens* Spores
Accuracy	0.830	0.825	0.840	0.855	0.940	0.925	0.950	0.960
Precision	0.824	0.828	0.847	0.845	0.949	0.913	0.959	0.950
Recall	0.840	0.820	0.830	0.870	0.930	0.940	0.940	0.970
Specificity	0.820	0.830	0.850	0.84	0.950	0.910	0.960	0.950
F1-Score	0.832	0.824	0.838	0.857	0.939	0.926	0.949	0.960

## Data Availability

Data are contained within the article.
